# BUILDING BRIDGES TO BETTER INTERGENERATIONAL RELATIONSHIPS

**DOI:** 10.1093/geroni/igad104.0939

**Published:** 2023-12-21

**Authors:** Tamar Shovali

**Affiliations:** Eckerd College, St. Petersburg, Florida, United States

## Abstract

Intergenerational relationships are necessary to prepare the workforce for careers in the field of gerontology. Intergenerational interaction and reflection have been shown to result in valuable skills, such as positive attitudes toward older adults, increased knowledge of aging and needs of older adults, genuine relationships with older adults and a greater appreciation for older generations, greater comfort with the idea of themselves aging, and high satisfaction when embedded into course curriculum. Intergenerational contact in the structure of undergraduate courses also has reciprocal benefits, such as recognizing commonalities, building appreciation and trust, and creating a sense of belonging between generations that can be lasting. Building on empirical evidence, an intensive three-week intergenerational relationships course for first year students using intentional integration with general education goals, while also training students in basic understanding of aging is described here. Students collaborated with the college’s Academy of Senior Professionals, a unique community of retired older adults engaged in lifelong learning, to develop a college-wide intergenerational event. Considerations, outcomes, and lessons learned will be reviewed.

